# Pathological hypertrophy and cardiac dysfunction are linked to aberrant endogenous unsaturated fatty acid metabolism

**DOI:** 10.1371/journal.pone.0193553

**Published:** 2018-03-01

**Authors:** Loreta Casquel De Tomasi, Dijon Henrique Salomé Campos, Paula Grippa Sant’Ana, Katashi Okoshi, Carlos Roberto Padovani, Gilson Masahiro Murata, Son Nguyen, Stephen C. Kolwicz, Antonio Carlos Cicogna

**Affiliations:** 1 Department of Internal Medicine, São Paulo State University, Botucatu, São Paulo, Brazil; 2 Mitochondria and Metabolism Center, Department of Anesthesiology & Pain Medicine, University of Washington, Seattle, Washington, United States of America; 3 Department of Biostatistics, São Paulo State University, Botucatu, São Paulo, Brazil; 4 Department of Biochemistry, University of São Paulo, São Paulo, São Paulo, Brazil; 5 Heart and Muscle Metabolism Laboratory, Health and Exercise Physiology Department, Ursinus College, Collegeville, Pennsylvania, United States of America; Texas A&M University Health Sciences Center, UNITED STATES

## Abstract

Pathological cardiac hypertrophy leads to derangements in lipid metabolism that may contribute to the development of cardiac dysfunction. Since previous studies, using high saturated fat diets, have yielded inconclusive results, we investigated whether provision of a high-unsaturated fatty acid (HUFA) diet was sufficient to restore impaired lipid metabolism and normalize diastolic dysfunction in the pathologically hypertrophied heart. Male, Wistar rats were subjected to supra-valvar aortic stenosis (SVAS) or sham surgery. After 6 weeks, diastolic dysfunction and pathological hypertrophy was confirmed and both sham and SVAS rats were treated with either normolipidic or HUFA diet. At 18 weeks post-surgery, the HUFA diet failed to normalize decreased E/A ratios or attenuate measures of cardiac hypertrophy in SVAS animals. Enzymatic activity assays and gene expression analysis showed that both normolipidic and HUFA-fed hypertrophied hearts had similar increases in glycolytic enzyme activity and down-regulation of fatty acid oxidation genes. Mass spectrometry analysis revealed depletion of unsaturated fatty acids, primarily linoleate and oleate, within the endogenous lipid pools of normolipidic SVAS hearts. The HUFA diet did not restore linoleate or oleate in the cardiac lipid pools, but did maintain body weight and adipose mass in SVAS animals. Overall, these results suggest that, in addition to decreased fatty acid oxidation, aberrant unsaturated fatty acid metabolism may be a maladaptive signature of the pathologically hypertrophied heart. The HUFA diet is insufficient to reverse metabolic remodeling, diastolic dysfunction, or pathologically hypertrophy, possibly do to preferentially partitioning of unsaturated fatty acids to adipose tissue.

## Introduction

Based on epidemiological evidence demonstrating reduced cardiovascular risk and mortality with consumption of diets high in unsaturated fatty acids [1,2], dietary intervention strategies remain an attractive therapeutic option to combat the metabolic and cardiac remodeling processes that occur in the hypertrophied heart. However, previous studies using various high fat diets in animal models have yielded mixed results. These studies have suggested that lipid metabolic pathways may be improved [3–6] following high fat diet administration but pathological remodeling remains unaffected [4,5] or attenuated [6–8]. In addition, cardiac function may be improved [6,9], impaired [3], or similar to chow fed animals [5,7]. The inconsistency in the data may be a consequence of several factors including: differences in the experimental models, dietary composition of the high fat diets utilized in the study, the time of initiation and duration of treatment, and the outcome measures analyzed.

Closer inspection of the literature reveals that in rodent models of left ventricular (LV) hypertrophy/dysfunction induced by pressure-overload [3–5,7], myocardial infarction [9], or sodium intake [6,8], most studies employed diets that were predominantly high in saturated fatty acids. Conversely, a diet rich in unsaturated fatty acids has been suggested to reduce plasma triglycerides, cardiac arrhythmias, sudden death, risk of ischemic heart disease and heart failure, and is therefore recommended to improve cardiovascular health [10]. In addition, administration of a diet rich in unsaturated fatty acids could provide the more appropriate ligand to stimulate fatty acid metabolism through activation of the peroxisome proliferator-activated receptor alpha (PPARα) pathway [11] and may attenuate or correct the metabolic and mechanical dysfunction that occurs during the pathological cardiac remodeling process. Therefore, we hypothesized that administration of a diet high in unsaturated fatty acids would have a beneficial effect on cardiac dysfunction, pathological remodeling, and lipid metabolism in the pressure-overloaded heart.

For this study, we selected the supra-valvar aortic stenosis model (SVAS) to promote gradual development of LV hypertrophy in young rats [12–15]. Initially, the pressure overload is mild and progressively increases as the animals grow in size, thus, recapitulating the clinical situation more closely. In our laboratory, echocardiographic studies showed that after 2 and 6 weeks of AS induction, rats developed LV hypertrophy, diastolic dysfunction and elevated systolic function [14,15]. Since there is a paucity of data evaluating the effect of high fat diet treatment on energy metabolism and cardiac function after the establishment of ventricular hypertrophy accompanied by diastolic dysfunction, the high-fat diet was administered 6 weeks after SVAS surgery in order to mimic a treatment model. Our results suggest that depletion of unsaturated fatty acids, specifically oleate and linoleate may be an additional hallmark of altered lipid metabolism in the hypertrophied myocardium. Provision of a high-unsaturated fatty acid diet does not normalize lipid metabolism, correct diastolic dysfunction, or attenuate pathological remodeling possibly due to a preferential partitioning of unsaturated fatty acids to adipose tissue storage.

## Materials and methods

### Animals

Male, *Wistar* rats (± 80g, 3 weeks old), were bred in-house, and kept in individual cages in a climate-controlled environment with a 12 h light/dark cycle and free access to food and water. All experiments conformed to the *Guide for the Care and Use of Laboratory Animals* published by the U.S. National Institutes of Health and was approved Botucatu Medical School Animal Research Ethics Committee (protocol 1094/14).

### Experimental design

Initially, rats (n = 50) underwent either supra-valvar aortic stenosis (SVAS) or sham surgery and fed standard chow. After 6 weeks, randomized animals were fed with a normolipidic diet (Sham-N, n = 13 and AS-N, n = 11) or high-unsaturated fatty acid (HUFA) diet (Sham-H, n = 12 and AS-H, n = 14) for 12 weeks. Animals in all groups were evaluated 12 weeks after the administration of the diets (i.e., 18 weeks after surgery).

### Supra-valvar aortic stenosis procedure

Aortic stenosis (AS) was induced surgically as described previously [13,14]. Rats (70–90 g) were anesthetized with intraperitoneal ketamine (60 mg/kg) and xylazine (10 mg/kg), and the heart was exposed via a median thoracotomy. Then, a silver clip (0.6 mm internal diameter) was placed on the ascending aorta at approximately 3 mm from its root. During the surgery, the rats received 1 ml of warm saline solution intraperitoneal and manually ventilated with positive pressure, on 100% oxygen. After the procedure, animals were kept warm until full consciousness was regained. Sham animals underwent the same procedure but without constriction of the aorta. After the surgery, all animals received an injection of meloxicam (5mg/kg) every 24 hours for 3 days. Rats were monitored twice per day for the first week and then at least twice per week for the remainder of the study. Post-operatively, rats were monitored for physical and clinical signs consistent with the AVMA Guidelines for Euthanasia. Animals that were monitored for edema, ascites, pleuro-pericardial effusion, and left atrial thrombus.

### Diets

**Fig 1:**
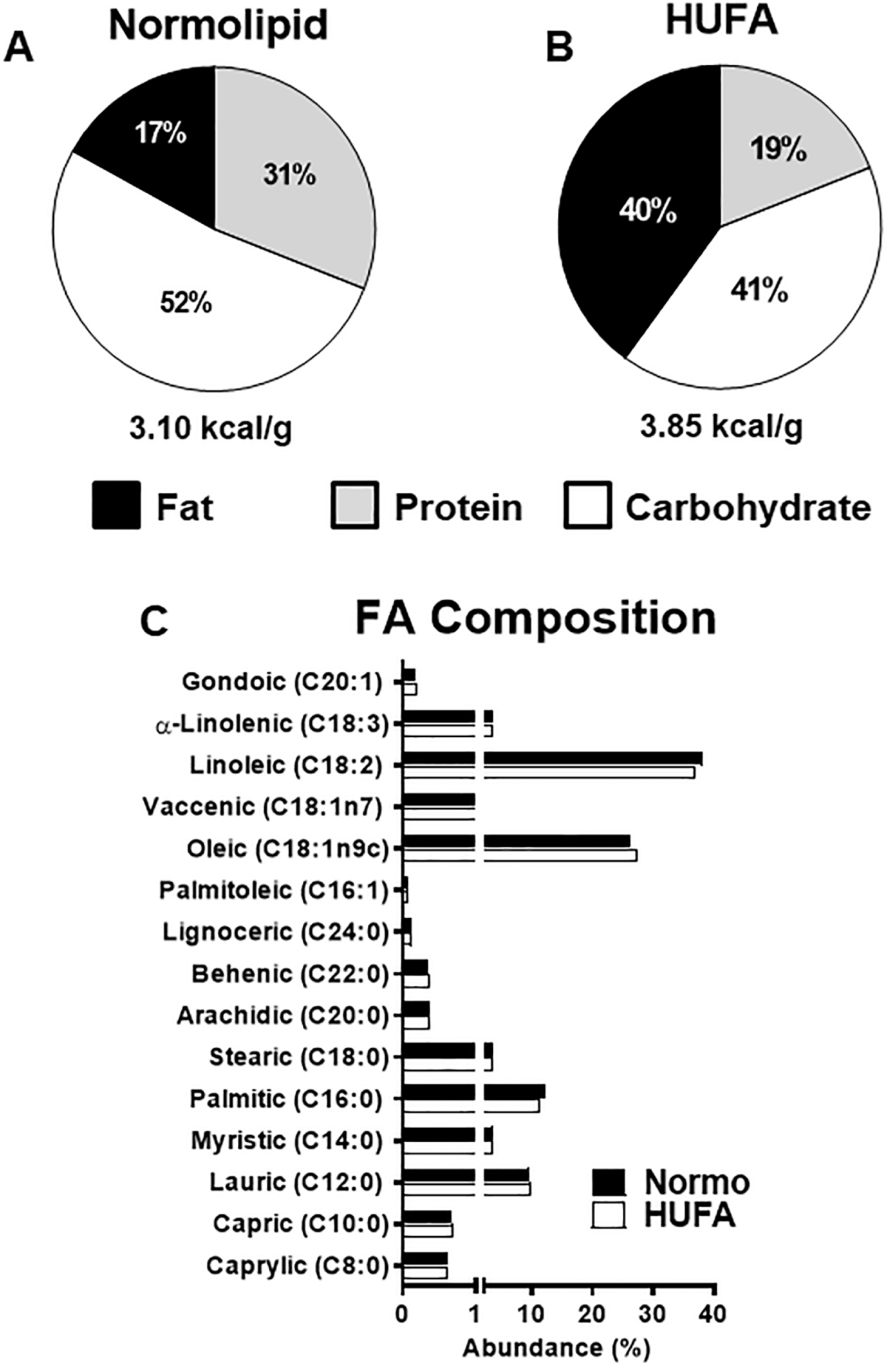
Caloric content and fatty acid composition of diets used in the study. (A-B) The percentage of total calories arising from fats (black), protein (grey), and carbohydrates (white) in the normolipid (Normo) and high unsaturated fat diet (HUFA). (C) The relative abundance of fatty acids in both diets. Fatty acids are organized by degree of unsaturation and carbon length.

Diets were custom manufactured (Biotron Zoocténica^®^, Rio Claro, SP, Brazil) using the following ingredients: soybean meal, soybean hull, corn, dextrin, soybean oil, palmitic oil, vitamin and mineral premix. The quantities were added to produce two different diets in lipid content, but with similar fatty acid composition and the same amount of carbohydrates in grams/100grams (Fig 1). Body weight and food consumption were monitored weekly. Caloric intake was calculated weekly as the average weekly food consumption (grams) times the dietary energetic density (kilocalories).

### Echocardiography

In-vivo cardiac function and morphometry was measured at 6 and 18 weeks post-surgery via echocardiography (Vivid S6, General Electric Medical Systems, Tirat Carmel, Israel) using a 5–11.5 MHz multi-frequency transducer. Rats were anesthetized by intraperitoneal injection of a mixture of ketamine (50 mg/kg) and xylazine (0.5 mg/kg). A two-dimensional parasternal short-axis view of the left ventricle (LV) was obtained at the level of the papillary muscles [16]. M-mode tracings were obtained from short-axis views of the LV at or just below the tip of the mitral-valve leaflets, and at the level of the aortic valve and left atrium. M-mode images of the LV were printed on a black-and-white thermal printer (Sony UP-890MD) at a sweep speed of 100 mm/s. All LV structures were manually measured by the same observer according to the leading-edge method of the American Society of Echocardiography [17]. Measurements reported are the average of at least five cardiac cycles from the M-mode tracings.

### Blood and tissue Harvest

At the end of 18 weeks, animals were fasted for 12–15 hours, anesthetized with sodium pentobarbital (50 mg/kg, IP) and killed via decapitation by trained and certified technician. Trunk blood was instantly collected and plasma was then separated by centrifugation at 1300 *g* for 10 minutes at 4°C, and stored at -80°C for subsequent analysis. The heart was removed, dissected, weighed, and frozen in liquid nitrogen. Adipose tissue was isolated from the epididymal, visceral, and retroperitoneal fat pads and weighed. Adiposity index was calculated from the sum of the individual fat pad weights:

(epididymal+visceral+retroperitoneal/(bodyweight)×100.

### Serum Metabolite analysis

Plasma non-esterified fatty acids and triacylglycerol concentrations were measured using colorimetric assays (Wako Chemicals, Richmond, VA, USA; and Bioclin, Belo Horizonte, MG, Brazil, respectively). Blood glucose concentration was measured using the Accu-Chek Go Kit glucose analyzer (Roche Diagnostics Brazil Ltd., São Paulo, Brazil).

### Real-Time PCR

Expression of cardiac genes involved in fatty acid oxidation was analyzed by Real-Time PCR. Total RNA was extracted from the left ventricle (LV) using Trizol (Invitrogen), according to the manufacturer’s instructions. The High Capacity cDNA reverse transcription kit for RT-PCR^®^ (Invitrogen, São Paulo, Brazil) was used for the synthesis of 20 μL complementary DNA (cDNA) from 1000 ng of whole RNA. The mRNA levels were determined by RT-qPCR using the following assays: PPARα Rn00566193_m1, PGC1α Rn00580241_m1, FAT/CD36 Rn00580728_m1, CPT1β Rn00566242_m1 and MCAD Rn00566390_m1 (Applied Biosystems). Quantitative measurements were made in the “Applied Biosystems StepOne Plus” detection system using the TaqMan qPCR commercial kit (Invitrogen) according to the manufacturer’s instructions. All assays were performed in triplicate. The mRNA of target genes was normalized to β-actin (assay Rn00667869_m1) and differences in expression were determined by the CT method described in the ABI user’s manual (Life Technologies).

### Metabolic enzymatic assays

Activities of key enzymes (i.e., phosphofructokinase (PFK), hexokinase (HK), lactate dehydrogenase (LDH), betahydroxyacyl CoA dehydrogenase (OHADH), and citrate synthase (CS), that participate in glucose and fatty acid metabolism were analyzed in the heart. LV samples were homogenized 1:20 (wt/vol) in 50 mM Tris-HCl, 1 mM EDTA, and protease inhibitor cocktail, pH 7.4, using a Polytron instrument (Kinematica, Littau-Lucerne, Switzerland). The lysate was centrifuged at 12000rpm for 10min at 4°C and the supernatant collected. All enzyme activities were determined at 25°C using a Spectra Max 250 microplate spectrophotometer (Molecular Devices, Sunnyvale, CA) and the assay buffer without the sample was used as blank. PFK was assayed in a buffer (pH 8.2) containing: (50mM Tris-HCl, 6.7mM MgCl_2_, 200mM KCl, 1mM beta-mercaptoethanol, 0.05% Triton X-100, 1mM ATP, 2mM AMP, 0.2mM NAD^+^, 0.9 units/ml aldolase, 0.16 units/ml glyceraldehyde 3-phosphate dehydrogenase, 9.6 units/ml triosephosphate isomerase, antimycin A and 3mM fructose-6-phosphate) and measured at 340 nm. HK was assayed in a reaction mixture containing 75mM Tris-HCl, 7.5mM MgCl_2_, 0.8mM EDTA, 1.5mM KCl, 4mM B-mercaptoethanol, 0.05% Triton X-100, 0.4mM NADP+, 2.5mM ATP, 1.4 units/ml glucose-6-phosphate dehydrogenase and 1.0mM glucose, pH 7.2; monitored at 340 nm. LDH was assayed in reaction mix containing 20mM Tris, 6.0mM pyruvate, 5.0mM NADH and monitored at 340nm. For OHADH, the reaction contained 100mM PBS pH 7.3, 0.45mM NADH and 0.1mM acetoacetyl CoA. CS activity was measured in reaction containing 100mM Tris-HCl, 1mM MgCl_2_, 1mM EDTA, 0.2 mM dithio-bis(2-nitrobenzoic acid), 0.3mM acetyl CoA and 0.5mM oxaloacetate, pH 8.2. The rate of change in absorbance was monitored at 412 nm (ε = 13.6 μmol · ml^−1^ · cm^−1^).

### Western blot analysis

Left ventricular tissue was homogenized in cold RIPA lysis buffer (Amresco, Solon, OH, USA) containing protease and phosphatase inhibitors (Roche, Indianapolis, IN, USA). The homogenate was centrifuged at 12000g for 20min at 4°C, and the supernatant was collected. Protein concentrations were determined using BCA Protein Assay kit (ThermoScientific, Wilmington, DE, USA). Samples (50μg) were subjected to SDS-polyacrylamide gel electrophoresis in polyacrylamide gels (6% or 10% depending on protein molecular weight). After electrophoresis, proteins were electro-transferred to nitrocellulose membrane (Bio-Rad Laboratories, Hercules, CA, USA). The blotted membranes were blocked with 5% nonfat dry milk in Tris-buffered saline/Tween-20 (25mM Tris, pH 7.5, 140mM sodium chloride, 3mM potassium chloride and 0.1% Tween-20) for 2 hours at room temperature. Membranes were then incubated overnight at 4°C with primary antibodies SERCA2 ATPase (ABR, Affinity BioReagents, Golden, CO, USA), phospholamban (Abcam, Cambridge, MA, USA), phospho–phospholamban (Ser16), phospho-phospholamban (Thr17) (Badrilla, Leeds, West Yorkshire, UK) and β-actin (Cell Signaling, Danvers, MA, USA). Binding of the primary antibody was detected with the use of peroxidase-conjugated secondary antibodies (anti-rabbit or anti-mouse IgG—Abcam, Cambridge, MA, USA) incubated for 1.5 hours at room temperature. Protein bands were visualized via chemiluminescent detection (Supersignal, Pierce, Rockford, IL, USA) in a western blot detection system (ImageQuant^™^ LAS 4000—GE Healthcare Life Sciences, Chalfont, UK), and quantified by densitometry using Image J Analysis software. Targeted bands were normalized to the expression of cardiac β-actin.

### Myosin heavy chain (MyHC) analysis

Cardiac MyHC isoforms were separated by discontinuous gel electrophoresis. Briefly, frozen samples of left ventricle were homogenized on ice in a solution containing 50 mM potassium phosphate buffer (pH 7.0), 300mM sucrose, 0.5mM dithiotreitol (DTT), 1mM ethylenediaminetetraacetic acid (EDTA), 0.3mM phenylmethylsulfonyl fluoride (PMSF), 10mM sodium fluoride and protease inhibitor cocktail (Sigma, St. Louis, MO, USA). Homogenates were centrifuged at 12000g for 20min at 4°C and total protein quantification was performed in supernatant aliquots using BCA Protein Assay kit (ThermoScientific, Wilmington, DE, USA). Samples were diluted to a final concentration of 1μg of protein/μL in a solution containing 65% (vol/vol) glycerol, 2.5% (vol/vol) β-mercaptoethanol, 1.15% (wt/vol) SDS, and 0.45% (wt/vol) Tris-HCl (pH 6.8). Then, 12ug of total protein was loaded into SDS-PAGE vertical slab gels (Hoefer, Holliston, MA, USA). The gels were run for 40 h at 20°C and constant voltage of 100 V. Protein bands for alpha and beta MyHC isoforms were visualized with Coomassie blue. Gels were digitally photographed with the Gel Logic 6000 Pro Imaging System (Carestream Health, Rochester, NY, USA), and analyzed for relative MyHC isoform percentages by densitometry using Image J Analysis software.

### Cardiac lipid analysis

Total lipids were extracted from heart tissue using a modified Folch method and solid phase extraction (SPE) protocol [18]. Frozen LV samples (20–30 mg wet weight) were homogenized in 2:1 chloroform:methanol. After centrifugation, the chloroform layer was removed and injected into a Bond Elut NH2 SPE column (Agilent Technologies, Santa Clara, CA) for the separation of triacylglycerol (TAG), diacylglycerol (DAG), free fatty acids (FFA) and phospholipids (PL). After drying under a nitrogen stream, residues were reconstituted in 2.5% H_2_SO_4_ in methanol. Samples were incubated at 80°C for 1 hour to convert to fatty acid methyl esters (FAMEs). After cooling, samples were washed twice with hexane to reclaim the FAMEs. FAMEs from the 4 different pools were analyzed by gas chromatography/mass spectrometry (GC/MS) using the Agilent 7890B gas chromatograph linked to an Agilent 5977A mass selective detector (Palo Alto, CA) operated at 70 eV, ion source temperature 260°C, mass range m/z 35–450. GC was performed using a 30 m x 0.25 mm I.D. column (HP-88, Agilent Technologies, Santa Clara, CA) with helium as carrier gas at a flow rate of 1 ml/min. The temperature gradient started with an initial temperature of 100°C, followed by a linear increase to 180°C at 20°C/min, and a slower linear increase to 220°C at 5°C/min. Identification of FAMEs was confirmed by a commercially available standard (47885-U Sigma-Aldrich, St. Louis, MO). Relative abundance was determined by the ratio of each fatty acid to sum of all fatty acids identified in the sample.

### Histology

Picrosirius red staining for assessment of cardiac fibrosis was performed as previously described [19]. In brief, LV transverse sections were fixed in 10% buffered formalin and embedded in paraffin as previously described. Sections of 1μm were cut from the tissue block and stained with hematoxylin and eosin, and with the collagen-specific stain picrosirius red (Sirius red F3BA in aqueous saturated picric acid). Images (40×) were collected using a camera attached to a Leica microscope (Leica Mikroskopie & Systems GmbH, Germany). Images were analyzed via Image J analysis software.

### Statistical analysis

Data are expressed as mean ± standard error of mean (SEM). Comparisons at the 6^th^ week were done by Student’s t-test and at the 18^th^ week by two-way ANOVA and Tukey post-hoc analysis. GraphPad Prism 7.0 was used for all statistical tests. *P* < 0.05 was accepted as statistically significant.

## Results

### Supra-valvar aortic stenosis induces cardiac hypertrophy and diastolic dysfunction

Six weeks after SVAS surgery, all rats underwent morphometric and functional analysis via echocardiography. As shown in Table 1, the AS group had significantly elevated ejection fraction (EF) and fractional shortening (FS) with significantly increased left ventricular posterior wall thickness (LVPW;d), relative wall thickness and LV mass index, suggestive of hyper-systolic function and cardiac hypertrophy. The AS group presented with decreased E/A ratios, in addition to, significantly increased left atrium (LA) diameter and LA to aorta ratio (LA/Ao), indicative of diastolic dysfunction (Table 1). As the SVAS surgery induced both cardiac hypertrophy and diastolic dysfunction as expected, rats in the AS groups were randomized to receive either the normolipidic or HUFA diet (Fig 1).

**Table 1:** Echocardiographic data 6 weeks post SVAS surgery.

	Sham	AS
**HR (bpm)**	326 ± 63	297 ± 24
**EF (%)**	91.0 ± 2.0	98.0 ± 1.0*
**FS (%)**	29.6 ± 3.4	37.1 ± 3.0*
**E wave (cm/s)**	80.8 ± 6.4	78.3 ± 14.7
**A wave (cm/s)**	52.2 ± 4.5	69.2 ± 21.7
**E/A ratio**	1.55 ± 0.06	1.18 ± 0.41*
**LVID;d (mm)**	7.21 ± 0.17	6.77 ± 0.13
**LVPW;d (mm)**	1.42 ± 0.03	1.72 ± 0.04*
**Relative wall thickness**	0.40 ± 0.04	0.50 ± 0.07*
**LV mass index (g/kg)**	2.25 ± 0.20	2.76 ± 0.49*
**LA (mm)**	4.94 ± 0.65	5.87 ± 0.71*
**LA/Ao**	1.42 ± 0.19	1.66 ± 0.29*

HR, heart rate; EF, ejection fraction; FS, fractional shortening; LVID;d, left ventricular internal dimension in diastole; LVPW; left ventricular posterior wall thickness in diastole; LA, left atrium diameter, LA/Ao: left atrium to aorta ratio.

*p<0.05 vs. sham. Sham, n = 8; AS, n = 25.

### High-unsaturated fat diet preserves body fat in rats after surgery

During the course of the 12-wk feeding period, food intake was monitored daily. Although food intake was lower in both sham and AS rats fed the HUFA diet compared chow groups, caloric intake was not different between the four groups (Table 2). AS surgery led to significant reduction in total body fat and adiposity index in normolipidic fed rats; however, this loss was prevented in AS animals fed HUFA (Table 2). Triglyceride and glucose levels were unchanged 18-wks post AS surgery in both diet groups; however, non-esterified free fatty acids concentration was decreased in AS-N but preserved in AS-H (Table 2).

**Table 2:** Physical characteristics and Serum Metabolite Concentratons.

	Normolipid		HUFA	
	Sham	AS	Sham	AS
**Body weight (g)**	447 ± 43	409 ± 30	447 ± 73	423 ± 31
**Food intake (g/day)**	29.1 ± 3.6	26.6 ± 2.8	22.1 ± 3.9 *	20.5 ± 2.2 ^#^
**Calorie intake (kcal/day)**	85.2 ± 10.7	78.1 ± 8.1	80.4 ± 14.2	74.6 ± 8.0
**Total body fat (g)**	19.6 ± 5.9	13.1 ± 3.6 *	23.4 ± 9.6	24.7 ± 4.8 ^#^
**Adiposity index**	4.61 ± 1.45	3.28 ± 0.75 *	5.13 ± 1.51	5.82 ± 1.00 ^#^
**Glucose (mg/dL)**	130 ± 9	125 ± 18	143 ± 18	138 ± 17
**Triacylglycerol (mg/dL)**	46.4 ± 16.1	39.5 ± 15.3	46.9 ± 14.2	46.8 ± 13.9
**NEFA (mM)**	0.35 ± 0.10	0.28 ± 0.03 *	0.35 ± 0.04	0.33 ± 0.08

NEFA: non-esterified fatty acids.

* p<0.05 vs. Sham-N;

^#^ p<0.05 vs. AS-N, (n = 11–14 each group.)

### Unsaturated fat diet does not attenuate cardiac hypertrophy or diastolic dysfunction

Echocardiography was performed on all rats at the end of the 12-wk feeding period (i.e., 18-wk post-surgery). Systolic function (EF and FS) remained significantly elevated in both AS groups compared to sham although the AS-H was slightly reduced compared to AS-N (Table 3). A wave velocity was significantly increased in both AS groups that resulted in a significant decrease in the E/A ratio (Table 3). Fibrosis, assessed by picrosirius red staining, was increased similarly in both AS surgery groups, consistent with diastolic dysfunction (S1 Fig). LVPW;d, relative wall thickness and left atrial dimensions were increased to a similar degree in both AS groups (Table 3), suggesting that cardiac hypertrophy was not attenuated or exacerbated by the HUFA. In support of the echocardiography data, the left and right ventricular weight as well as atrial weight was increased to a similar degree in both AS-N and AS-H groups (Table 4). Taken together, these results demonstrate that feeding a diet high in unsaturated fat does not improve diastolic dysfunction or attenuate pathological cardiac growth in rats subjected to SVAS surgery.

**Table 3:** Echocardiographic data 12 weeks after high unsaturated fat diet treatment.

	Normolipid		HUFA	
	Sham	AS	Sham	AS
**HR (bpm)**	293 ± 58	272 ± 32	306 ± 37	280 ± 45
**EF (%)**	92.0 ± 3.0	98.0 ± 1.0 *	92.0 ± 2.0	96.0 ± 3.0 * ^#^
**FS (%)**	30.3 ± 3.2	37.3 ± 2.9 *	29.3 ± 2.3	32.2 ± 4.1 * ^#^
**E wave (cm/s)**	74.0 ± 9.1	77.3 ± 16.6	75.2 ± 9.6	75.0 ± 8.2
**A wave (cm/s)**	49.2 ± 8.3	62.2 ± 13.7 *	46.8 ± 10.5	60.0 ± 15.5 *
**E/A ratio**	1.52 ± 0.16	1.29 ± 0.30 *	1.69 ± 0.19	1.28 ± 0.42 *
**Relative wall thickness**	0.37 ± 0.03	0.57 ± 0.06 *	0.39 ± 0.04	0.57 ± 0.05 *
**LVID;d (mm)**	7.84 ± 0.19	7.25 ± 0.22	7.45 ± 0.15	7.08 ± 0.18
**LVPW;d (mm)**	1.46 ± 0.02	2.04 ± 0.02 *	1.46 ± 0.03	2.05 ± 0.04 *
**LV mass index (g/kg)**	1.72 ± 0.22	2.71 ± 0.42 *	1.59 ± 0.09	2.50 ± 0.39 *
**LA (mm)**	4.99 ± 0.57	6.41 ± 0.85 *	5.23 ± 0.39	6.17 ± 0.72 *
**LA/Ao**	1.29 ± 0.13	1.67 ± 0.18 *	1.31 ± 0.08	1.55 ± 0.23 *

HR, heart rate; EF, ejection fraction; FS, fractional shortening; LVID;d, left ventricular internal dimension in diastole; LVPW; left ventricular posterior wall thickness in diastole; LA, left atrium diameter, LA/Ao: left atrium to aorta ratio.

*p<0.05 vs. respective sham.

^#^ p<0.05 vs. AS-N, (n = 11–14 each group).

**Table 4:** Cardiac mass analysis.

	Normolipid		HUFA	
	Sham	AS	Sham	AS
**LV (g)**	0.81 ± 0.11	1.16 ± 0.12 *	0.78 ± 0.09	1.16 ± 0.17 *
**RV (g)**	0.28 ± 0.07	0.25 ± 0.04	0.23 ± 0.03 *	0.27 ± 0.03 *
**AT (g)**	0.11 ± 0.02	0.17 ± 0.04 *	0.10 ± 0.02	0.16 ± 0.03 *
**Tibia (cm)**	4.24 ± 0.07	4.18 ± 0.08	4.24 ± 0.12	4.22 ± 0.10
**LV/tibia (g/cm)**	0.19 ± 0.02	0.28 ± 0.03 *	0.18 ± 0.02	0.27 ± 0.04 *
**RV/tibia (g/cm)**	0.07 ± 0.02	0.06 ± 0.01	0.05 ± 0.01 *	0.06 ± 0.01 *
**AT/tibia (g/cm)**	0.02 ± 0.004	0.04 ± 0.009 *	0.02 ± 0.005	0.04 ± 0.007 *

LV: left ventricle weight; RD: right ventricle weight; AT: atrium weight; Tibia: tibia length.

* p<0.05 vs sham, (n = 11–14 each group).

### High-unsaturated fatty acid diet does not prevent changes in glycolytic enzyme activity or fatty acid oxidation genes

**Fig 2:**
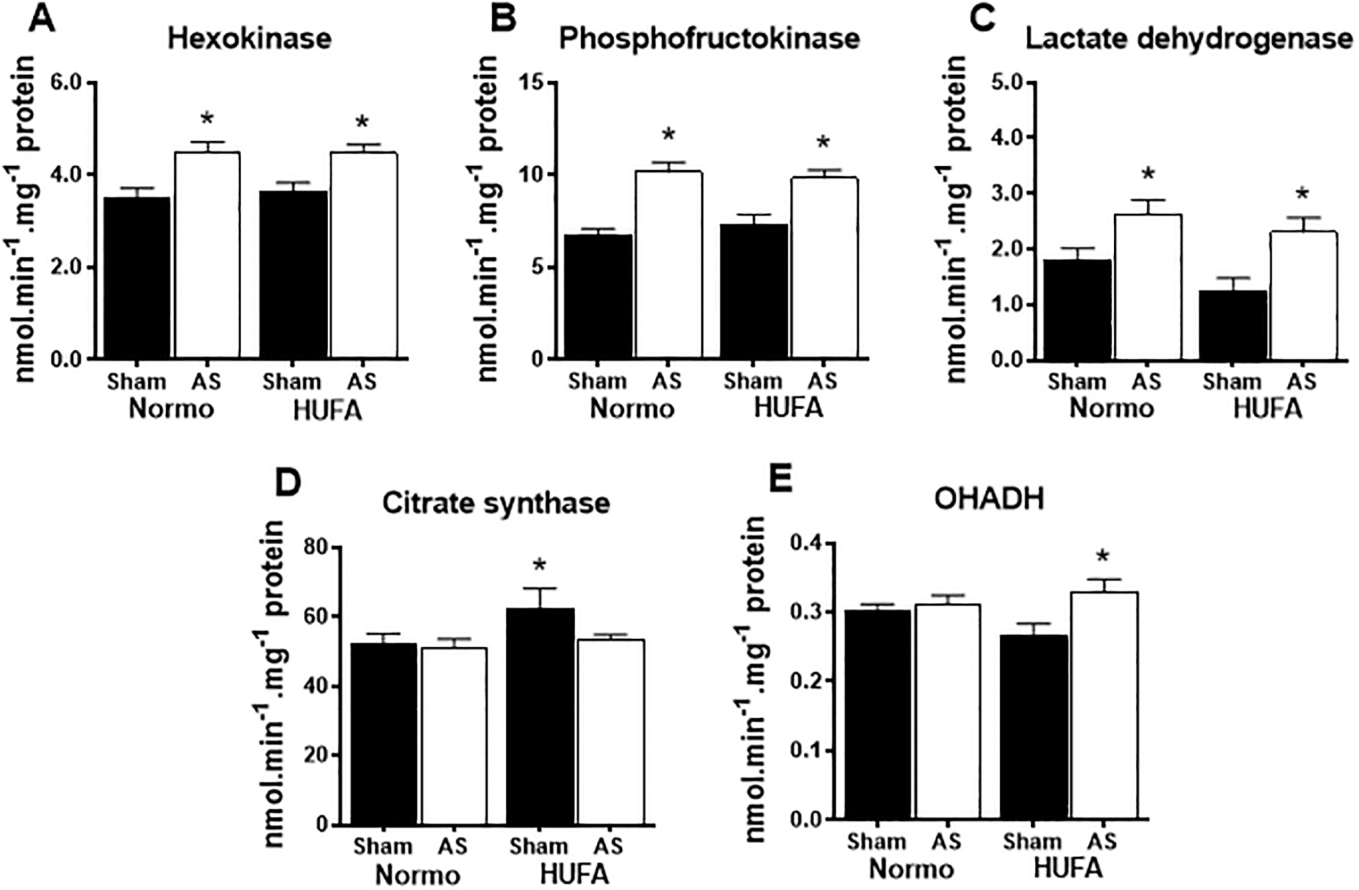
Unsaturated fatty acid diet does not prevent upregulation of glycolytic enzyme activity in hypertrophied hearts. (A-C) Activities of hexokinase, phosphofructokinase and lactate dehydrogenase in heart lysates from sham and AS groups fed normolipidic (Normo) or high unsaturated fat diet (HUFA); (D) Citrate synthase activity; (E) Beta hydroxy-acyl CoA dehydrogenase (OHADH). * p<0.05 vs. respective sham (n = 9–13 per group).

**Fig 3:**
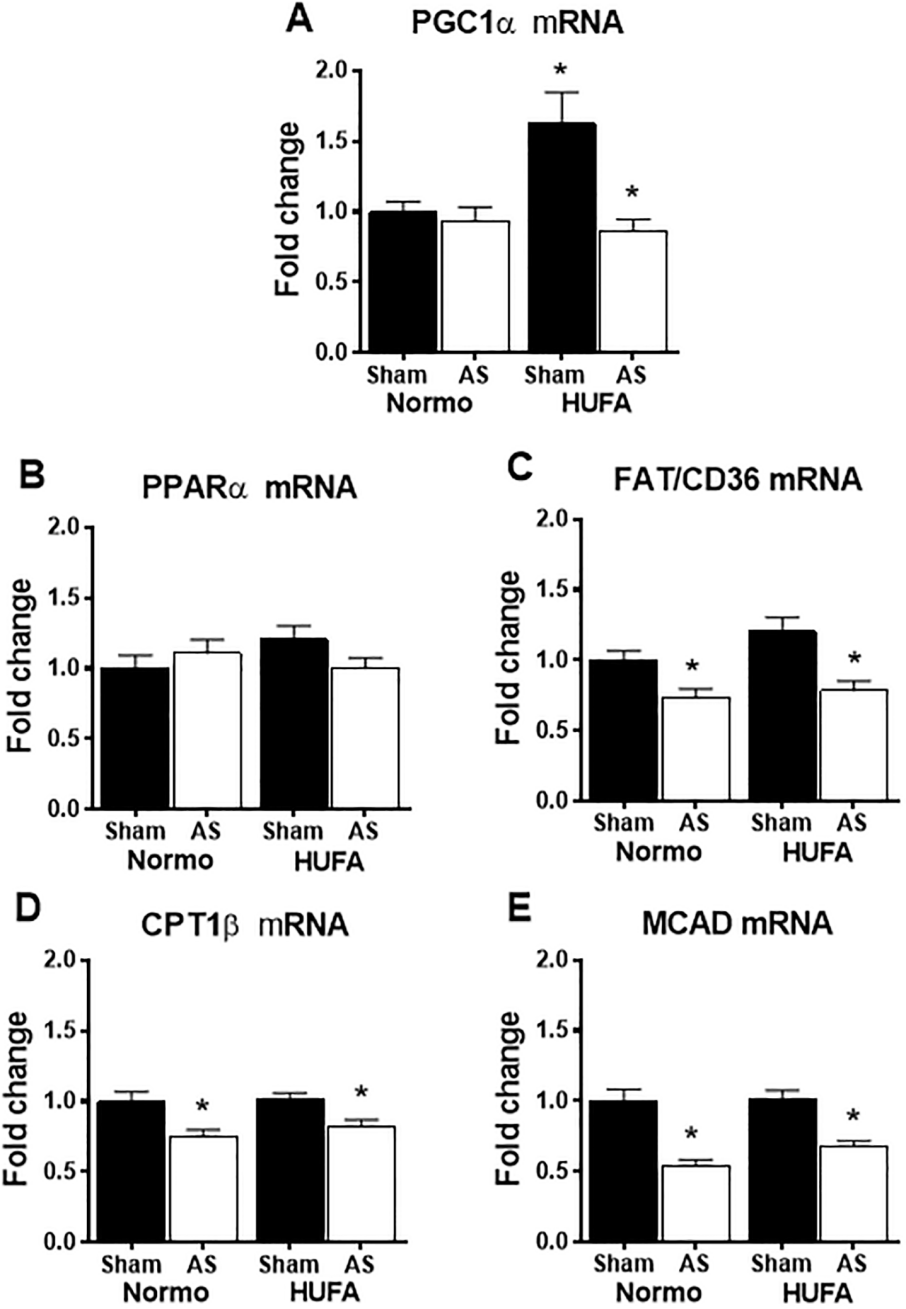
Unsaturated fat diet does not reverse down-regulation of fatty acid oxidation (FAO) related genes. Expression of cardiac genes involved in fatty acid oxidation from sham and AS groups fed normolipidic (Normo) or high-unsaturated fat diet (HUFA). (A) PGC1α: peroxisome proliferator-activated receptor gamma coactivator 1-alpha; (B) PPARα: peroxisome proliferator-activated receptor alpha; (C) FAT/CD36: fatty acid translocase; (D) CPT1β: carnitine palmitoyltransferase I beta; (E) MCAD: medium-chain acyl-CoA dehydrogenase. Data presented as fold-change from Sham-Normo. * p<0.05 vs. respective sham, (n = 9–11 per group).

Hypertrophied hearts are known to shift to a fetal metabolic profile, where decreased fatty acid oxidation is countered by an upregulation in glycolytic activity. Consistent with this, significant increases were observed in the activities of hexokinase, phosphofructokinase, and lactate dehydrogenase in both AS-N and AS-H hearts (Fig 2A–2C). Interestingly, citrate synthase activity was increased by the unsaturated fat diet in sham animals, but remained unchanged in both AS groups (Fig 2D). Beta-hydroxyacyl coenzyme-A dehydrogenase (OHADH) activity was not affected by AS surgery but was mildly increased in AS hearts fed the HUFA (Fig 2E). Similar to citrate synthase activity, a significant upregulation of PGC1α gene expression was observed in sham hearts fed the HUFA with no change noted in the AS groups (Fig 3A). Gene expression of PPARα remained unaffected in AS groups; however, the downstream targets FAT/CD36, CPT1B, and MCAD were significantly down regulated in both control and HUFA hearts subjected to AS surgery (Fig 3B–3E). In total, these results show that provision of a high-unsaturated fat diet to hearts 6 weeks post-AS surgery is not sufficient to reverse the upregulation glycolytic enzyme activity or decrease in fatty acid oxidation genes, consistent with the fetal metabolic profile in hypertrophied hearts.

### Decreases in calcium handling proteins are not restored by unsaturated fat diet

**Fig 4:**
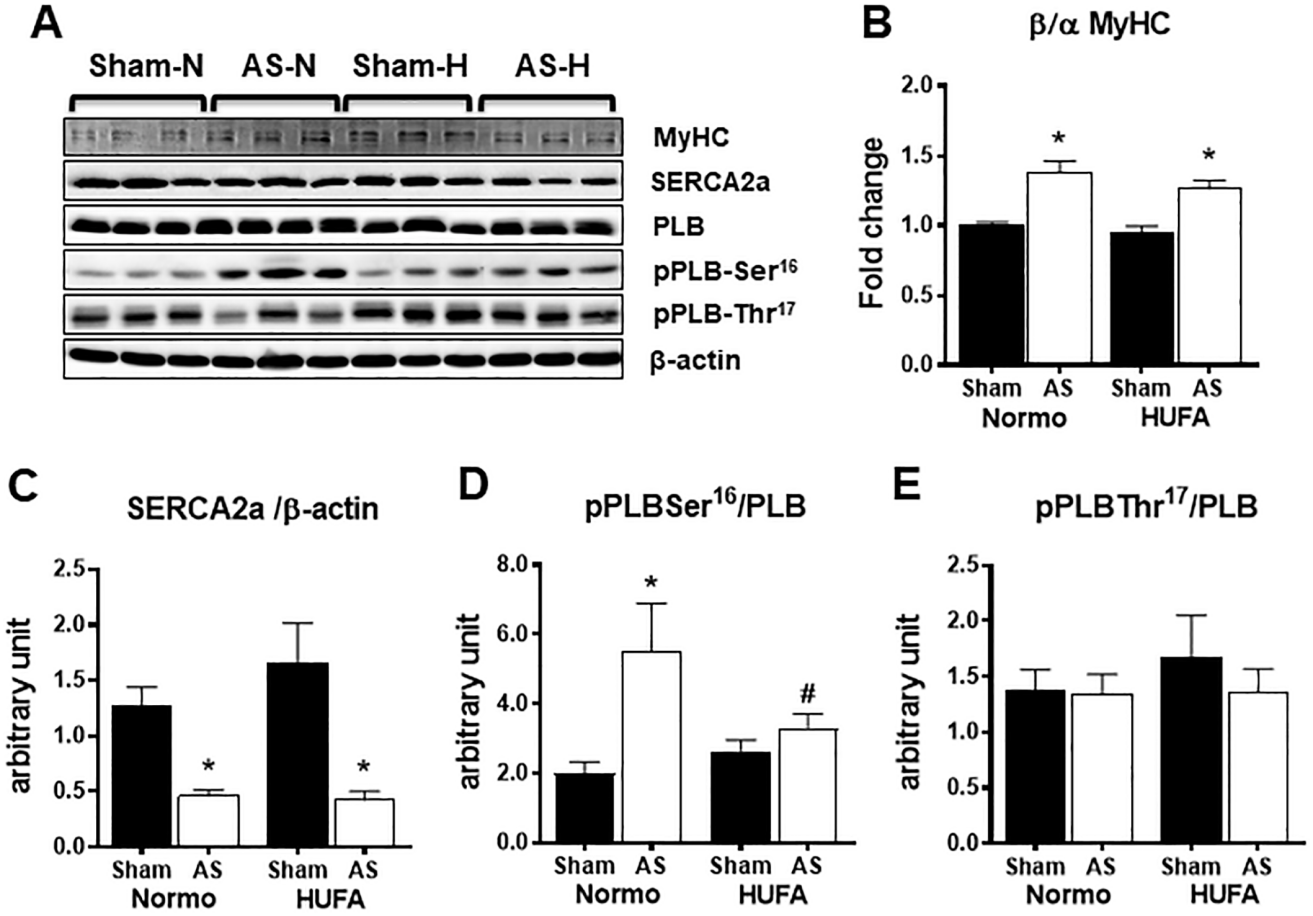
Altered myosin heavy chain or calcium handling protein expression are not affected by high-unsaturated fat diet. Representative image of protein bands from (A) myosin heavy chain (MyHC), sarco/endoplasmic reticulum Ca2+-ATPase (SERCA2a), phospholamban (PLB), phosphorylated PLB at serine-16 (pPLB-Ser^16^), phosphorylated PLB at threonine-17 (pPLB-Thr^17^), and beta-actin (β-actin). (B) Quantification of the β-MyHC to α-MyHC isoforms. (C) Quantification of the SERCA2 normalized to β-actin. (D) Quantification of pPLB-Ser^16^ normalized to PLB. (E) Quantification of pPLB-Thr^17^ normalized to PLB. Data reported as fold change relative to Sham-Normo. * P <0.05 vs. respective sham; # <0.05 vs. AS-N, n = 6 each group.

Consistent with previous reports [20,21], the ratio of the beta to alpha isoform of myosin heavy chain was increased in the hypertrophied myocardium without a significant dietary effect (Fig 4A and 4B). In addition, AS surgery led to a 50% decrease in SERCA2a protein expression that was not improved with the unsaturated fat diet (Fig 4A and 4C). Since increased phosphorylation of phospholamban (PLB) at Serine 16 (S16), mediated by protein kinase A, and PLB at Threonine 17 (T17), mediated by calmodulin-dependent kinase II (CaMKII) has been suggested to contribute to altered calcium responsiveness to beta-adrenergic signaling in cardiac hypertrophy [22,23], the phosphorylation status of these PLB sites were assessed. Increased phosphorylation of PLB at S16, but not T17, was observed in AS-N that was attenuated in AS-H group (Fig 4D and 4E). In all, these data show that AS induces alterations in calcium handling proteins, consistent with pathological hypertrophy, that are not corrected by the HUFA diet.

### Hypertrophied hearts have less unsaturated fatty acids in endogenous lipid pools

**Fig 5:**
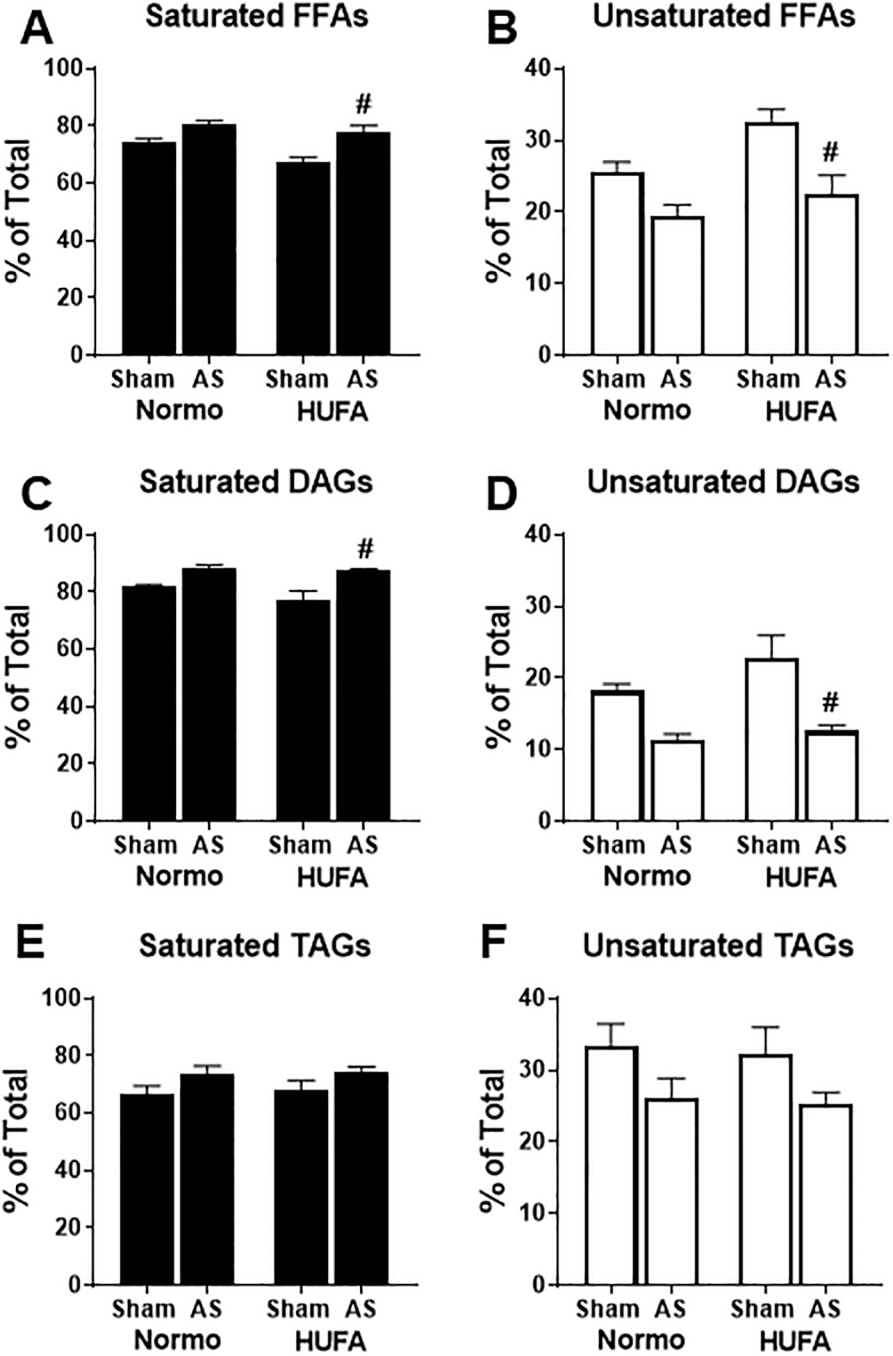
Abundance of saturated and unsaturated fatty acids of cardiac lipid pools is not altered with diet in hypertrophied hearts. The abundance of saturated and unsaturated fatty acids was determined in lipid extracts from cardiac tissue in Sham and AS hearts fed normolipidic (Normo) or high-unsaturated fat diet (HUFA). Lipid extracts were separated into: (A) saturated cytosolic free fatty acids (FFAs); (B) unsaturated FFAs; (C) saturated fatty acids from diacylglycerol (DAG); (D) unsaturated fatty acids from DAG; (E) saturated fatty acids from triacylglycerol (TAG); (F) unsaturated fatty acids from DAG. All saturated fatty acids and unsaturated fatty acids, as determined by GC-MS analysis, were summed and the percentage of total fatty acids was calculated. Data are reported as mean ± SEM for each group. ^#^ p<0.05 vs. Sham-HUFA, n = 5 each group.

The HUFA diet failed to elicit any beneficial effects on metabolism, in-vivo function, or hypertrophic growth in AS hearts. Therefore, we further probed lipid metabolism by profiling the endogenous cardiac lipid pools using GC-MS. Fig 5 shows the relative proportion of saturated and unsaturated fatty acids detected within the various endogenous lipid pools of free fatty acids (FFAs), diacylglycerol (DAG), and triacylglycerol (TAG). Overall, saturated fatty acids account for approximately 70–80% of the FFA (Fig 5A) and DAG (Fig 5C) pools with less saturated fatty acids appearing in the TAG (~60%, Fig 5E) and PLs (~50%, S2 Fig) pools in sham hearts exposed to the normolipidic diet. The HUFA diet had a tendency to increase unsaturated fatty acids by ~25% in both the FFA and DAG pools after 12 weeks of feeding in sham animals (p<0.10) while the TAG pool remained relatively stable (Fig 5A–5F). Following AS surgery, the abundance of unsaturated fatty acids in the FFA pool trended lower by ~25% (p<0.10) and remained significantly lower in HUFA-fed AS rats (Fig 5B). A similar pattern was observed in the DAG pool where unsaturated fatty acids were reduced by ~40% in both AS-Normo and AS-HUFA hearts (Fig 5D). The HUFA diet did not affect the abundance of unsaturated fatty acids within the TAG pool in sham animals; however, AS surgery led to an approximate 20% reduction of unsaturated fats in TAGs in both groups (Fig 5F). Cardiac PLs remained stable regardless of surgery or dietary intervention (S2 Fig). These data suggest that 12 weeks of HUFA can cause a mild change in the proportion of unsaturated fatty acids within the unstressed myocardium. However, in the hypertrophied heart induced by SVAS surgery, HUFA is unable to mitigate the depletion of unsaturated fats from the endogenous cardiac lipid pools.

### Unsaturated fat diet does not restore depletion of oleic and linoleic acid in AS hearts

**Fig 6:**
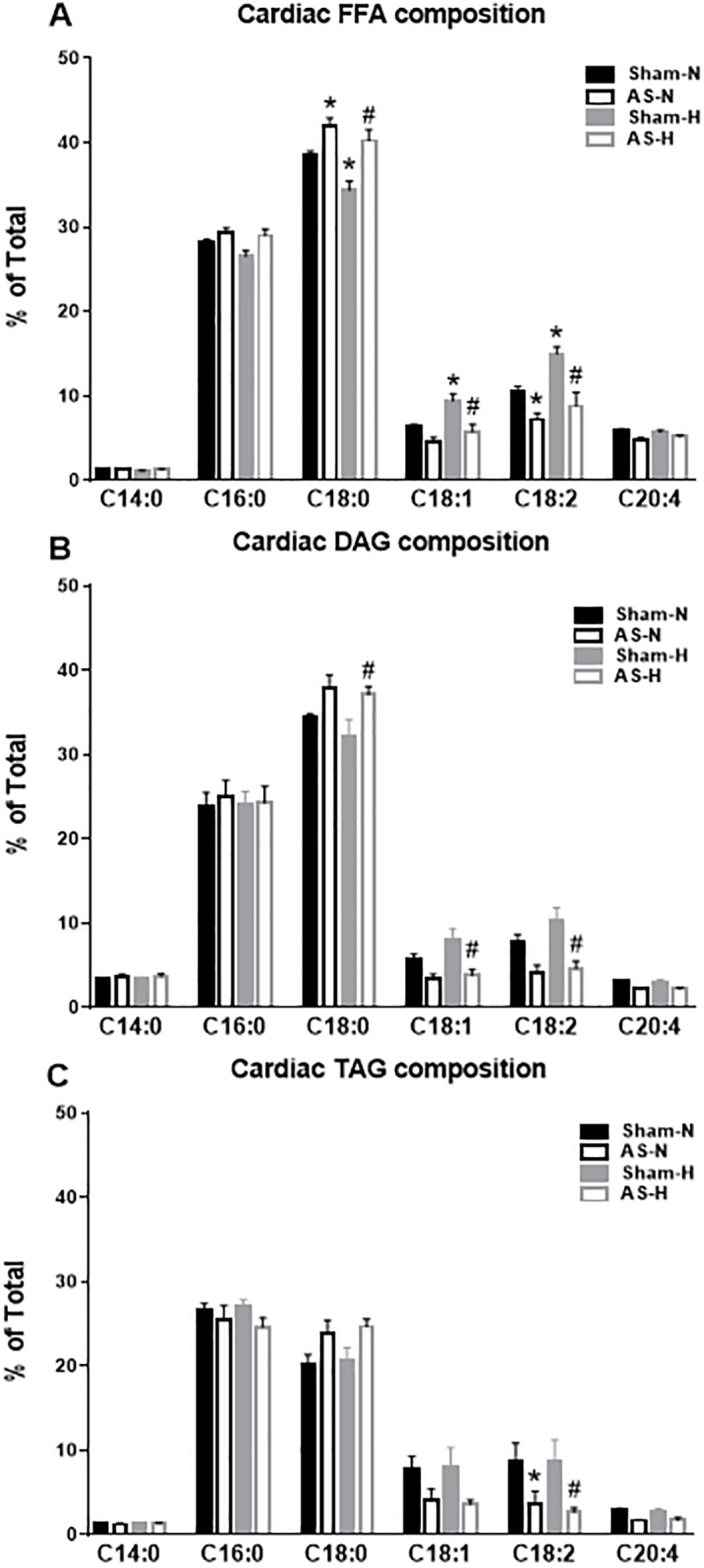
Decreased abundance of oleic and linoleic acid in hypertrophied hearts is not restored by diet. The relative abundance of individual fatty acids present in lipid extracts from cardiac tissue in Sham and AS hearts fed normolipidic (Normo, Sham-N and AS-N) or high unsaturated fat diet (HUFA, Sham-H and AS-H). Lipid extracts were separated into: (A) total cytosolic free fatty acids (FFAs); (B) diacylglycerol (DAG); and (C) triacylglycerol (TAG). Data is reported as relative abundance as determined by GC-MS analysis. * *p*<0.05 vs. Sham-N; # *p*<0.05 vs. Sham-H, (n = 5 each group).

To gain additional insight into the loss of unsaturated fats, we examined the fatty acid composition of the individual endogenous cardiac lipid pools. As shown in Fig 6, palmitate (C16:0) and stearate (C18:0) were the two most abundant fatty acids within the FFA, DAG, and TAG pools. This was also true of the PLs fraction with the addition of arachidonate and docosahexaenoate. (S3 Fig). In sham hearts, the HUFA diet caused a significant increase in both oleate (C18:1) and linoleate (C18:2) in the FFA pool but not within the DAG and TAG pools (Fig 6A–6C). In the FFA, DAG, and TAG pools, the relative abundance of C18:1 was decreased by ~30–50% in AS-N hearts and was at significantly lower levels in the FFA and DAG pools of AS-H hearts (Fig 6A–6C). Likewise, the abundance of C18:2 was significantly reduced by ~30–60% in FFA, DAG (p = .1036), and TAG pools as a result of AS surgery in normolipidic fed rats, which remained significantly lower in AS rats fed a HUFA (Fig 6A–6C). Despite changes in other lipid pools, the fatty acid composition of cardiac PLs was not affected by either surgery or diet (S3 Fig). These data suggest that 12 weeks of HUFA, with a high abundance of oleate (C18:1) and linoleate (C18:2) has a mild effect on the fatty acid composition of these unsaturated fatty acids in the FFA pool of healthy hearts, but not in hypertrophied hearts. Moreover, these data implicate potential alterations in lipid metabolism, specifically relating to unsaturated fatty acids (i.e., C18:1 and C18:2), in the pathological hypertrophied myocardium.

## Discussion

The major finding of the present study is that rat hearts subjected to supra-valvar aortic stenosis (SVAS) developed significant hypertrophy, diastolic dysfunction, and are prone to depletion of the unsaturated fatty acids, oleate and linoleate, within the endogenous cardiac lipid pools, potentially identifying this as an additional feature of abnormal lipid metabolism in pathological hypertrophy. Remarkably, a diet high in unsaturated fatty acids does not prevent the myocardial depletion of oleate and linoleate in this model. In total, our data demonstrate that a 3-month dietary intervention of high-unsaturated fat is insufficient to combat diastolic dysfunction in the hypertrophied myocardium. However, our data suggest that abnormal lipid partitioning, particularly relating to the unsaturated fats, linoleate and oleate, exists in the hypertrophied heart, which may be an additional hallmark of aberrant lipid metabolism.

Several previous studies attempted to rescue deficient fatty acid oxidation in pressure-overload hypertrophy rodent models using a high fat diet intervention that resulted in inconsistent conclusions. The majority of these studies started the dietary intervention shortly (i.e., within 1 week) after the surgery, mostly employing a high fat diet that contained predominantly saturated fat [5,7–9]. To account for this, we selected a diet that was rich in unsaturated fats, which has been suggested to be a beneficial dietary strategy to decrease cardiovascular disease risk and mortality [1,2]. In this regard, our diet would be consistent with dietary recommendations for the prevention and treatment of heart disease from the American Heart Association [10]. However, our results do not support consumption of unsaturated fatty acids, starting 6 weeks after SVAS surgery, as a successful secondary prevention strategy for cardiac dysfunction and pathological remodeling.

Unfortunately, our findings do not support the idea that diets high in unsaturated fats are beneficial for correcting cardiac dysfunction in the hypertrophied myocardium. However, the diet was only provided for 3 months so we cannot rule out that a longer duration of treatment would be more suitable. One notable change that occurred in the SVAS animals that received the HUFA diet was the preservation of body weight and adipose tissue mass. This could be a salient factor, particularly in regards to the “Obesity Paradox”, where mortality is reduced in obese patients with heart failure [24]. Although this phenomenon has recently been scrutinized [25], the long-term effect of the HUFA diet on cardiovascular mortality in our experimental model warrants further investigation.

In this work, we hypothesized that the increased supply of unsaturated fatty acids would lead to the reactivation of PPARα, thus, normalizing lipid uptake and oxidation and correcting metabolic and mechanical dysfunction in hypertrophied hearts. Our data show that genes related to lipid uptake and oxidation (CD36, CPT1β, MCAD) remain down regulated in the hypertrophied myocardium despite chronic feeding of unsaturated fatty acids. Previous studies found that rat hearts subjected to transverse aortic constriction (TAC) had decreased cardiac function and endogenous fatty acid oxidation in the Langendorff preparation when palmitate was supplemented in the perfusate [11,26]. Interestingly, provision of oleate, instead of palmitate, to the isolated TAC hearts improved parameters of contractility and relaxation and improved endogenous FAO, which was mediated by restoration of PPARα target genes [11]. Based on these reports, the inability of a chronic high-unsaturated fatty acid supply to improve cardiac function and/or lipid metabolism in our study is quite disappointing. An obvious explanation is the experimental setting, where in the isolated perfused heart, a concentrated amount of oleate was provided for a short time (i.e., ~60min). In this regard, the acute stimulatory effect of oleate could be the overwhelming factor. Oleate represented only ~30% of all fatty acids in the diet, and although was delivered for 12 weeks, it was dispersed throughout multiple organ systems. Therefore, a concentration and/or time dependent mechanism might be critical in the observed effects of the unsaturated fat, oleate, to augment cardiac function and lipid metabolism.

An essential question remains as to why the high-unsaturated fatty acid diet was incapable of affecting cardiac parameters, especially as epidemiological and basic science research would suggest otherwise. Perhaps, a better understanding of both cardiac and systemic lipid partitioning would make things clearer. Our lipid profiling of cardiac tissue shows that the unsaturated fats, linoleate and oleate, which represent over 60% of the total dietary fatty acid content, do not appear in the myocardium in ratios consistent with the exogenous supply. This becomes even more apparent in the hypertrophied heart as both of these fatty acids are decreased in multiple endogenous pools. Speculation could arise as to whether the hypertrophied heart preferentially oxidizes these particular fatty acids, which has been suggested to occur in the whole body of large animals and humans [27–29]. As the composition of both oleate and linoleate were well preserved within the cardiac phospholipids, another possibility is that these fatty acids are redirected into maintaining phospholipid integrity. It has been suggested that unsaturated fats within the phospholipid membrane, particularly linoleate, are prone to degradation within the stressed myocardium [30,31]. However, this would not explain the deficiencies of oleate and linoleate within the cardiac lipid pools of the hypertrophied hearts fed the high fat diet. Since the SVAS animals fed with the HUFA diet maintained body weight and fat mass, the increased exogenous supply of unsaturated fatty acids might be preferentially stored in the adipose tissue, rather than the heart. Reports in the literature lend support to this possibility. Two reports demonstrated that greater gains in body weight and fat mass occurred when mice were fed a high fat diet predominant in unsaturated versus saturated fats [32,33]. Furthermore, studies in rats [34] and humans [35–37] show that adipose tissue has a stronger correlation to the dietary profile than serum or other tissues. In total, our study raises additional questions about the partitioning of specific lipids in both the healthy and diseased heart [38] and suggest that additional studies are needed to unravel the complexities of lipid metabolism in cardiac pathologies.

Several other factors need to be considered regarding the findings in the present study. We employed the SVAS model, which induces a gradual pressure-overload as the animal ages [12–15] where in other models using aortic constriction, an abrupt change in the pressure gradient occurs [3–5,7]. Clearly, this could contribute to source of variation among studies. However, studies in coronary artery ligation [9] and salt-induced hypertension [6,8] also yield mixed results. Comparison outcomes across multiple studies is problematic as many different diets have been utilized and the macronutrient profile and composition of fatty acids are not always easily discernable. In this regard, reduction of nutrients such as carbohydrates may result in high fat diets that are more consistent with ketogenic diets that may confound results. Furthermore, additional understanding is needed regarding the uptake and partitioning of lipids due their carbon length and degree of saturation [38] so broad statements regarding the effects of saturated or unsaturated fatty acids should be expanded to identify the specific fatty acid.

In summary, the SVAS surgery model induces robust pathological hypertrophy, diastolic dysfunction, and altered lipid metabolism, highlighted by a depletion of unsaturated fatty acids within the endogenous cardiac lipid pools. Although a high content of oleate and linoleate was present in the high-unsaturated diet, it fails to restore the depleted oleate and linoleate content in hypertrophied hearts. This may be due to a preferential uptake of these fatty acids by the adipose tissue, which explains the inability of the diet to attenuate pathological hypertrophy, correct diastolic dysfunction, or normalize lipid metabolism. Overall, these findings hint towards abnormal lipid partitioning, specifically involving mono-unsaturated and polyunsaturated fatty acids, as an additional signature of maladaptive lipid metabolism in the hypertrophied myocardium.

## Supporting information

S1 Fig SVAS surgery increases fibrosis similarly in normolipidic and HUFA fed rats. Fibrosis was assessed by picrosirius red staining in cardiac sections and quantified by Image J. (A) Representative images from each of the four groups. (B) Percentage of fibrosis determined by the ratio of stained to unstained tissue. Data are reported as mean ± SEM for each group. * P <0.05 vs. respective sham, n = 3.
(TIF)

S2 Fig Abundance of saturated and unsaturated fatty acids of cardiac phospholipids is not altered with diet in hypertrophied hearts. The abundance of saturated and unsaturated fatty acids was determined in phospholipid extracts from cardiac tissue in Sham and AS hearts fed normolipidic (Normo) or high-unsaturated fat diet (HUFA). (A) The relative abundance of all saturated fatty acids. (B) The relative abundance of all unsaturated fatty acids. All saturated fatty acids and unsaturated fatty acids, as determined by GC-MS analysis, were summed and the percentage of total fatty acids was calculated. Data are reported as mean ± SEM for each group, n = 5.
(TIF)

S3 Fig Fatty acid composition of cardiac phospholipids. The relative abundance of individual fatty acids present in phospholipid extracts from cardiac tissue in Sham and AS hearts fed normolipidic (Normo, Sham-N and AS-N) or high unsaturated fat diet (HUFA, Sham-H and AS-H). Data is reported as relative abundance as determined by GC-MS analysis. (n = 5 each group). C14:0, myristic acid; C16:0, palmitic acid; C18:0, stearic acid; C18:1, oleic acid; C18:2, linoleic acid; C20:4, arachidonic acid; C22:6, docosahexaenoic acid.
(TIF)
