# Supplementary figures and images for: Pathological hypertrophy and cardiac dysfunction are linked to aberrant endogenous unsaturated fatty acid metabolism
Source: PLoS One. 2018 Mar 1;13(3):e0193553. doi: 10.1371/journal.pone.0193553 (PMC5832311; doi:10.1371/journal.pone.0193553)

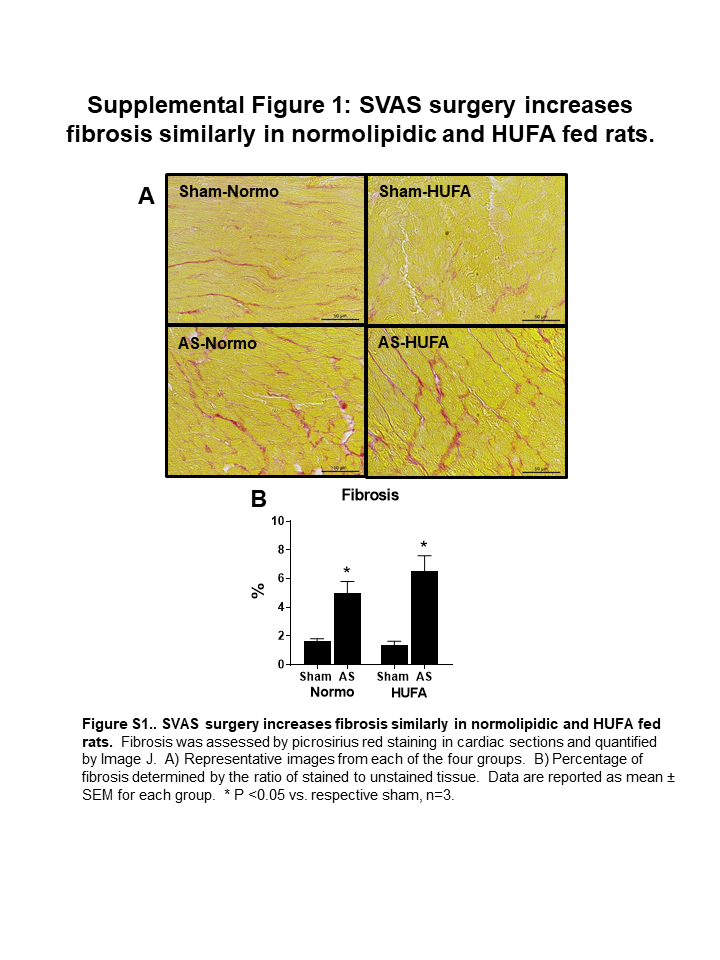

Supplement: S1 Fig — Fibrosis was assessed by picrosirius red staining in cardiac sections and quantified by Image J. (A) Representative images from each of the four groups. (B) Percentage of fibrosis determined by the ratio of stained to unstained tissue. Data are reported as mean ± SEM for each group. * P <0.05 vs. respective sham, n = 3. (TIF) [file pone.0193553.s001.TIF]

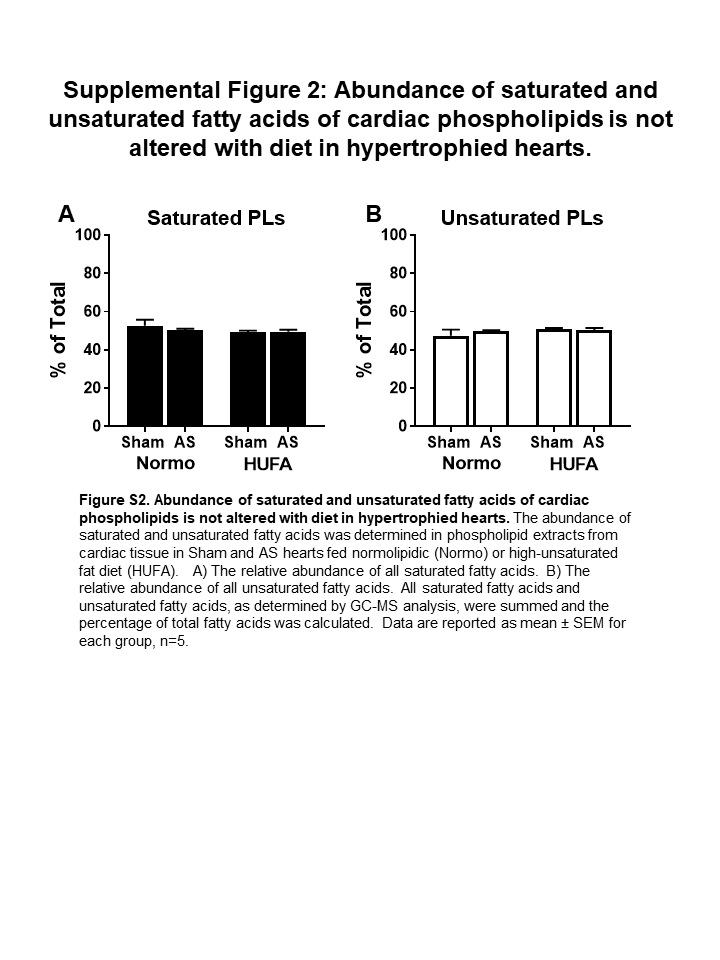

Supplement: S2 Fig — The abundance of saturated and unsaturated fatty acids was determined in phospholipid extracts from cardiac tissue in Sham and AS hearts fed normolipidic (Normo) or high-unsaturated fat diet (HUFA). (A) The relative abundance of all saturated fatty acids. (B) The relative abundance of all unsaturated fatty acids. All saturated fatty acids and unsaturated fatty acids, as determined by GC-MS analysis, were summed and the percentage of total fatty acids was calculated. Data are reported as mean ± SEM for each group, n = 5. (TIF) [file pone.0193553.s002.TIF]

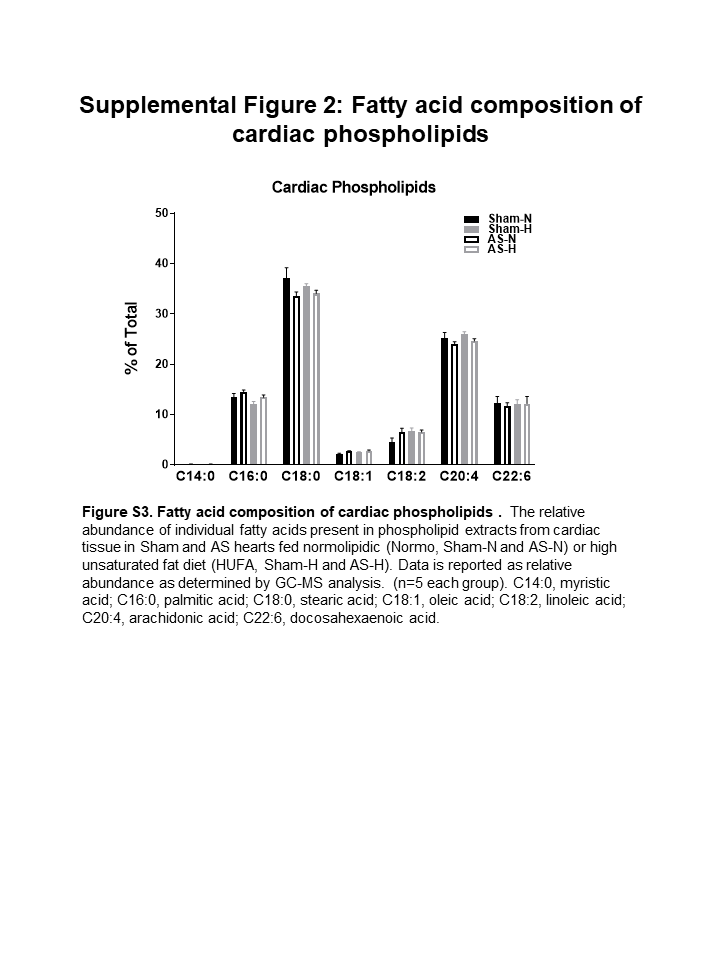

Supplement: S3 Fig — The relative abundance of individual fatty acids present in phospholipid extracts from cardiac tissue in Sham and AS hearts fed normolipidic (Normo, Sham-N and AS-N) or high unsaturated fat diet (HUFA, Sham-H and AS-H). Data is reported as relative abundance as determined by GC-MS analysis. (n = 5 each group). C14:0, myristic acid; C16:0, palmitic acid; C18:0, stearic acid; C18:1, oleic acid; C18:2, linoleic acid; C20:4, arachidonic acid; C22:6, docosahexaenoic acid. (TIF) [file pone.0193553.s003.TIF]
